# Measuring patient safety culture in Taiwan using the Hospital Survey on Patient Safety Culture (HSOPSC)

**DOI:** 10.1186/1472-6963-10-152

**Published:** 2010-06-07

**Authors:** I-Chi Chen, Hung-Hui Li

**Affiliations:** 1 College of Management, Yuan Ze University, TaoYuan, Taiwan; 2 Department of Health Care Administration, Oriental Institute of Technology, Taipei County, Taiwan

## Abstract

**Background:**

Patient safety is a critical component to the quality of health care. As health care organizations endeavour to improve their quality of care, there is a growing recognition of the importance of establishing a culture of patient safety. In this research, the authors use the Hospital Survey on Patient Safety Culture (HSOPSC) questionnaire to assess the culture of patient safety in Taiwan and attempt to provide an explanation for some of the phenomena that are unique in Taiwan.

**Methods:**

The authors used HSOPSC to measure the 12 dimensions of the patient safety culture from 42 hospitals in Taiwan. The survey received 788 respondents including physicians, nurses, and non-clinical staff. This study used SPSS 15.0 for Windows and Amos 7 software tools to perform the statistical analysis on the survey data, including descriptive statistics and confirmatory factor analysis of the structural equation model.

**Results:**

The overall average positive response rate for the 12 patient safety culture dimensions of the HSOPSC survey was 64%, slightly higher than the average positive response rate for the AHRQ data (61%). The results showed that hospital staff in Taiwan feel positively toward patient safety culture in their organization. The dimension that received the highest positive response rate was "Teamwork within units", similar to the results reported in the US. The dimension with the lowest percentage of positive responses was "Staffing". Statistical analysis showed discrepancies between Taiwan and the US in three dimensions, including "Feedback and communication about error", "Communication openness", and "Frequency of event reporting".

**Conclusions:**

The HSOPSC measurement provides evidence for assessing patient safety culture in Taiwan. The results show that in general, hospital staffs in Taiwan feel positively toward patient safety culture within their organization. The existence of discrepancies between the US data and the Taiwanese data suggest that cultural uniqueness should be taken into consideration whenever safety culture measurement tools are applied in different cultural settings.

## Background

Patient safety is crucial to the health care quality. According to the adverse event reports in Taiwan, there were 14,945 cases reported in 2007, and many of these accidents and deaths could have been prevented [1]. Those accidents and failures were extremely costly both for the patients and for the health care system. As health care industries strive to improve, there is a growing recognition of the importance of establishing a culture of patient safety.

Patient safety in health care organizations has received much attention following the Institute of Medicine report "*To Err Is Human: Building a Safer Health System" *[2]. The concept of patient safety culture originated from research of safety in hazardous industries. Cox and Cox [3] defined safety culture as the collection of attitudes, beliefs, perceptions, and values that employees share in relation to safety. Similarly, Nieva & Sorra [4] defined patient safety culture as the product of individual and group values, attitudes, perceptions, competencies, and patterns of behaviour that determine the commitment to, and the style and proficiency of, an organization's safety management. An organization with a 'safety culture' is open and fair with staff when incidents occur, learns from mistakes, and rather than blaming individuals, looks at what went wrong in the system [3,4].

Patient safety culture is a complex framework which involves different dimensions that guides many discretionary behaviours of patient safety [4,5]. According to the Agency of Healthcare Research and Quality (AHRQ) [6], patient safety culture requires an understanding of the values, beliefs, and norms about what is important in an organization and what attitudes and behaviours related to patient safety are supported, rewarded, and expected. Therefore, it is important for health care organizations to assess their culture regarding patient safety in order to improve patient safety within the health care process.

The Hospital Survey on Patient Safety Culture (HSOPSC) of AHRQ [6] is a tool for assessing the safety culture of hospitals as a whole, or for specific units within the hospitals. HSOPSC has good psychometric criteria testing, including item analysis, exploratory factor analysis, confirmatory factor analysis, and inter-correlation and reliability analysis [7-10]. HSOPSC has been tested on a large sample, and has good supporting documentation [8,9,11]. HSOPSC also has been used in different countries including the United States, Canada, and Belgium, and has been translated into different languages. In addition to HSOPSC, AHRQ also provides a database of 382 hospitals in the US participating in the HSOPSC survey. The database consists of data from 108,621 respondents who completed the survey, including nurses, physicians, technicians, pharmacists and administrators in 2007.

The main objective of this research was to use the HSOPSC measurement tool to evaluate patient safety culture in Taiwan's hospitals and attempt to provide explanation of some of the phenomena in patient safety culture that are unique in Taiwan. The findings of this study should provide health care organizations in Taiwan a better understanding about patient safety culture in Taiwan's hospitals.

## Methods

### HSOPSC Questionnaire

HSOPSC was pilot tested, revised, and then released in November 2004 by AHRQ [6]. It was designed to measure 12 factors (dimensions) of patient safety culture. The HSOPSC questionnaire contains 42 items which mostly use the 5-point Likert response scale of agreement ("Strongly disagree" to "Strongly agree") or frequency ("Never" to "Always"). The survey measures:

(A) Seven unit-level aspects of safety culture

(1) Supervisor/manager expectations and actions promoting safety (4 items)

(2) Organizational learning-continuous improvement (3 items)

(3) Teamwork within units (4 items)

(4) Communication openness (3 items)

(5) Feedback and communication about error (3 items)

(6) Nonpunitive response to error (3 items)

(7) Staffing (4 items)

(B) Three hospital-level aspects of safety culture

(8) Hospital management support for patient safety (3 items)

(9) Teamwork across hospital units (4 items)

(10) Hospital handoffs and transitions (4 items)

(C) Two outcome variables

(11) Overall perceptions of safety (4 items)

(12) Frequency of event reporting (3 items)

The HSOPSC questions were translated into Chinese by one translator with background in patient safety research. The translation was then reviewed by a task group including eight professional experts from the medical, nursing, and patient safety fields.

For construct validity, the authors used the principal component analysis extraction method and the Varimax rotation method, the total variance explained by the 12 factors covered by HSOPSC was 61.57 percent (KMO = 0.868, *p *= 0.000). According to Fleming [9], the reliability expressed as Cronbach's α for the AHRQ data ranged from 0.63 to 0.84, whereas for the data in this research, the Cronbach's α ranged from 0.51 to 0.84, slightly lower than the AHRQ data, which implied that the consistency of the responses on each survey item for the data in this study is less than for the AHRQ data.

### Sample and data collection

Random sampling was used to survey a wide range of hospital staffs throughout Taiwan, including physicians representing each department, nurses representing each clinical nursing unit, and non-clinical staff. There are in total 23 medical centers, 20 regional hospitals, and 306 community hospitals in Taiwan. Of the 306 community hospitals, 23 are teaching hospitals. Only teaching hospitals were included in this study. A total of 50 hospitals (out of 68 teaching hospitals) were randomly selected for the survey, including 16 medical centers, 18 regional hospitals, and 16 community hospitals. For each hospital, a sample of 20 staff members was chosen randomly by a research coordinator in the hospital, which consisted of about 6 physicians, 12 nurses, and 2 administrators. The sample roughly represented all professional groups within a proportional allocation in Taiwan. The total sample size for the survey was 1000, including 300 physicians, 600 nurses, and 100 administrators. The investigation was conducted from January 2006 to February 2008.

To ensure the privacy of the respondents, the survey was strictly anonymous. Also, to allow for confidentiality, respondents were asked to put their completed questionnaire in a sealed envelope and the envelopes are collected by the research coordinator and then returned directly to the researchers. Formal consent to participate the survey was granted by the management board of each hospital. Formal ethical approval was not needed for this study according to Taiwan's law.

After receiving the completed questionnaires, a preprocessing step was applied to remove incomplete or invalid data. The exclusion criteria used were similar to [6,10]: (1) no entire section completed; (2) fewer than half the items answered; or (3) all items answered the same. After removing incomplete questionnaires, a total of 788 respondents (230 physicians, 478 nurses, and 80 administrators) from 42 hospitals had successfully completed the questionnaire. Therefore, the final response rate for the survey was 78.8%.

### Data analysis

This study used SPSS 15.0 for Windows and Amos 7 to perform the statistical analysis. First, descriptive statistics of the demographic characteristics of respondents, characteristics of hospitals, and the average percentage of positive responses on patient safety culture were computed. The average percentage of positive responses, defined as the average of the item-level percent positive responses within a HSOPSC dimension, represented positive reaction toward patient safety culture. Second, descriptive statistics and *t*-test were used to explore the differences between the data of this study and the 2007 AHRQ database, and also used to examine the differences in average positive response rate between the supervisors and non-supervisors within the data. Thirdly, confirmatory factor analysis (CFA) of SEM was performed to justify the adequacy of the HSOPSC assessment on the data in Taiwan. Lastly, construct reliability (CR) and internal correlation were tested to assist in better understanding of the measurement and concept of HSOPSC in Taiwan. CR also provided an indication of the internal consistency of the 12 dimensions. Internal correlation, on the other hand, showed the discriminant validity among the 12 dimensions.

## Results

In the following sections, we first show the demographic statistics for the respondents taking the survey. Next, we present the results of the HSOPSC survey at three different levels: the unit level, the hospital level, and the outcome level. The comparison between our data and the AHRQ data are also given. Following that are results comparing the differences in average positive response rate between the supervision group and non-supervision group. Finally, the results of applying HSOPSC in Taiwan are given.

### Demographic statistics

A total of 788 respondents from 42 hospitals (10 medical centers, 16 regional hospitals and 16 community hospitals) in Taiwan completed the survey. As shown in Table 1, about 29.2% (230) of the respondents were physicians across different departments, 60.6% (478) were nurses, and 10.2% (80) were administrators, which included clerks, secretaries, and managers. The average age of the respondents was 35 years old, and most of them were female (73.7%). They had worked an average of one to five years in their hospital. The largest percentage of physician respondents worked in medicine (60.8%), followed by surgery (19.6%) and cross unit (19.6%). Most of the nurses worked in general wards (36.4%), followed by cross department (27.0%), and then outpatient department (19.4%). The majority of the administrators did not have direct contact with the patients (92.5%). Among the respondents, 38.8% (306) were in a supervisory position. Since the government and hospital authority in Taiwan gave an impetus to patient safety, many of the hospital staffs (78.8%) received patient safety training programs within the hospital, but only 45.7% (360) received training programs outside the hospital. Furthermore, 96.2% of the hospitals had their own patient safety reporting mechanism, and 87.6% of them also had a specific department in charge with patient safety affairs. Table 1 also shows the demographic statistics separately for the supervisors and non-supervisors. As shown, majority (63.1%) of the supervisors were physicians. Comparing with the non-supervision group, the supervision group had higher education level (95.4% had college degree or higher versus 76.3%) and had longer working experience (50.6% had worked more than 5 years versus 30.1%) in hospitals. On the other hand, the non-supervision group had higher percentage in receiving patient safety training inside hospital (81.7%), and it consisted mostly female (92.3%).

**Table 1:** Demographic characteristics of respondents.

	Overall (%)	Supervisor (%)	Non -supervisor (%)		Overall (%)	Supervisor (%)	Non-supervisor (%)
*Hospital level*				*Work unit/department*			
Medical center hospital	189 (24.0%)	54 (17.7%)	135 (28.0%)	Physician			
Regional hospital	293 (37.2%)	120 (39.2%)	173 (35.9%)	Surgery ^a^	45 (19.6%)	38 (19.7%)	7 (19.0%)
Community hospital	306 (38.8%)	132 (43.1%)	174 (36.1%)	Medicine ^b^	140 (60.8%)	124 (64.2%)	16 (43.2%)
*Gender*				Cross unit	45 (19.6%)	31 (16.1%)	14 (37.8%)
Male	207 (26.3%)	170 (55.6%)	37 (7.7%)	Nurse			
Female	581 (73.7%)	136 (44.4%)	445 (92.3%)	Surgery ^a^	96 (20.1%)	27 (37.5%)	69 (17.0%)
*Staff position*				Obstetrics/ baby room	5 (1.0%)	0 (0%)	5 (1.2%)
Physician	230 (29.2%)	193 (63.1%)	37 (7.7%)	General ward	174 (36.4%)	25 (34.7%)	149 (36.7%)
Nurse	478 (60.6%)	72 (23.5%)	406 (84.2%)	Outpatient department	85 (17.8%)	3 (4.2%)	82 (20.2%)
Administrator	80 (10.2%)	41 (13.4%)	39 (8.1%)	Cross unit	118 (24.7%)	17 (23.6%)	101 (24.9%)
*Education level*				Administrator			
High school	26 (3.3%)	4 (1.3%)	22 (4.6%)	Indirect to patient ^c^	74 (92.5%)	37 (94.9%)	37 (90.2%)
Junior college	102 (12.9%)	10 (3.3%)	92 (19.1%)	Direct to patient ^d^	6 (7.5%)	2 (5.1%)	4 (9.8%)
College/university	597 (75.8%)	241 (78.8%)	358 (74.3%)				
Master	59 (7.5%)	47 (15.3%)	10 (2.0%)				
PhD	4 (0.5%)	4 (1.3%)	0 (0%)				
*Working time in hospital*				*Working hours per week*			
Less than 1 year	81 (10.3%)	17 (5.6%)	64 (13.3%)	Less than 20 hours	18 (2.3%)	12 (3.9%)	6 (1.2%)
1 to 5 years	407 (51.6%)	134 (43.8%)	273 (56.6%)	20 to 39 hours	108 (13.7%)	57 (18.6%)	51 (10.6%)
6 to 10 years	151 (19.2%)	67 (21.9%)	84 (17.4%)	40 to 59 hours	573 (72.7%)	193 (63.1%)	380 (78.8%)
11 to 15 years	87 (11.0%)	51 (16.7%)	36 (7.5%)	60 to 79 hours	63 (8.0%)	31 (10.1%)	32 (6.7%)
16-20 years	34 (4.3%)	20 (6.5%)	14 (2.9%)	80 to 99 hours	11 (1.4%)	5 (1.7%)	6 (1.2%)
21 years or more	28 (3.6%)	17 (5.5%)	11 (2.3%)	More than 100 hours	15 (1.9%)	8 (2.6%)	7 (1.5%)
*Patient safety training (in hospital)*				*Patient safety training (outside hospital)*			
Yes	621 (78.8%)	227 (74.2%)	394 (81.7%)	Yes	360 (45.7%)	177 (57.8%)	183 (38.0%)
No	167 (21.2%)	79 (25.8%)	88 (18.3%)	No	428 (54.3%)	129 (42.2%)	299 (62.0%)
*Adverse event report system set-up*				*Patient safety department set-up*			
Yes	758 (96.2%)	293 (95.8%)	465 (96.5%)	Yes	690 (87.6%)	278 (90.8%)	412 (85.5%)
No	9 (1.1%)	6 (2.0%)	3 (0.6%)	No	35 (4.4%)	11 (3.6%)	24 (5.0%)
Not sure	21 (2.7%)	7 (2.2%)	14 (2.9%)	Not sure	63 (8.0%)	17 (5.6%)	46 (9.5%)
*Level of position*							
Supervisor	306 (38.8%)	-	-				
Non-supervisor	482 (61.2%)	-	-				

a: Include surgery, ER and ICU.

b: Include internal, medicine, obstetrics, pediatrics, family medicine, and psychiatry.

c: Include information, human resource management, finance, secretary, and general affairs.

d: Include front desk, medical record department, and medical affairs.

### Unit-level aspects of patient safety culture

The unit-level aspects of patient safety culture represent the perception of respondents on patient safety culture within their department and unit. Table 2 shows the average percentage of positive responses for each of the 12 dimensions that HSOPSC measures, for both the 2007 AHRQ data and the data from this study (Taiwan). The results are sorted in descending order based on the AHRQ data, and the *t-*test results between the AHRQ data and Taiwan data are shown in the last column. The unit-level aspect of patient safety culture covers items 1, 2, 4, 6, 7, 10, and 12 in Table 2. The average percentage of positive responses for "Teamwork within units" is 94% in Taiwan, which is much higher than that reported by the AHRQ (78%). The results indicate that most of the respondents in this study feel supportive and respected in their unit or work place, and they are more likely to cooperate and coordinate with their co-workers. For the "Supervisor/manager expectations and actions promoting safety" dimension, the average percentage of positive responses for Taiwan is 83%, which is also higher than the AHRQ data (74%). The differences between the AHRQ data and the Taiwan data for both dimensions are significant different in a statistical sense.

**Table 2:** Average positive response rate for the HSOPSC results for Taiwan and AHRQ data.

HSOPSC Dimension	AHRQ	Taiwan	*p*-value
		
	Average Positive Response (%)	Average Positive Response (%)	
1. Teamwork within units	78%	94%	0.009***
2. Supervisor/manager expectations & actions promoting patient safety	74%	83%	0.026**
3. Hospital management support for patient safety	69%	62%	0.6467
4. Organizational learning -- continuous improvement	69%	84%	0.002***
5. Overall perceptions of safety	63%	65%	0.958
6. Feedback & communication about error	62%	59%	0.723
7. Communication openness	61%	58%	0.772
8. Frequency of event reporting	59%	57%	0.819
9. Teamwork across hospital units	57%	72%	0.002***
10. Staffing	55%	39%	0.012**
11. Hospital Handoffs & transitions	45%	48%	0.398
12. Nonpunitive response to error	43%	45%	0.847

***Significant different at α = 0.01, ** Significant different at α = 0.05.

The "Organizational learning--continuous improvement" dimension of patient safety culture represents a learning culture in which mistakes lead to positive changes and changes are evaluated for effectiveness. In Taiwan, most of the respondents agreed that their hospitals had constructive activities to improve patient safety culture. The percentage of positive responses for organizational learning in Taiwan is significantly higher than in the AHRQ data. However, for the "Feedback and communication about error" and "Communication openness" dimensions, the positive response rates for Taiwan are slightly lower than the AHRQ, although the differences are not significant.

In the past, when failure or error occurred, it usually resulted in punishing people instead of acknowledging the problem existed. The "Nonpunitive response to error" dimension measures to what extent the hospital staff feels that their mistakes are not held against them, and the mistakes are not kept in their personnel file. For both Taiwan and the AHRQ data, the positive response rate for this item is lower than 50%, and it also has one of the lowest scores among the twelve dimensions of patient safety culture.

The last unit-level patient safety culture dimension is "Staffing"; it shows whether a health care unit has adequate staff allocation to handle the workload and whether the working hours are appropriate for providing the best care for the patients. The percentage of positive responses for this item is the lowest for patient safety culture in this survey, and it is significant different from the AHRQ data.

### Hospital-level aspects of patient safety culture

The hospital-level aspects of patient safety culture cover items 3, 9, and 11 in Table 2. The "Hospital management support for patient safety" dimension is an indication of whether the management team provides a work climate that promotes patient safety. In Taiwan, the positive response rate for this item is 62%, which is lower than the AHRQ data (69%). For the "Teamwork across hospital units" dimension, on the other hand, the positive response rate for Taiwan (72%) is significantly higher than that for the AHRQ data (57%). Hospital workers in Taiwan seem to have better cooperation and coordination across different units or departments.

Medical problems and accidents may occur during shift changes. Therefore, unhindered handoff and transition is desirable to assure patient safety in the hospital. However, most respondents in Taiwan and the US feel that hospitals are not doing enough and the average percentage of positive responses for both surveys on this item are below 50%.

### Outcome-level aspects of patient safety culture

The outcome-level measurements of patient safety culture include "Overall perceptions of safety" and "Frequency of event reporting" (items 5 and 8 in Table 2). Overall perception of patient safety culture is an indication of good procedures and systems for preventing errors and the lack of patient safety problems. The percentage of positive response for Taiwan for this item is 65%, a little higher than the AHRQ result (63%). For the frequency of event reporting factor, the positive response rate is 56% for Taiwan, and 59% for the AHRQ data, but the difference is not significant.

### Supervision group versus non-supervision group

As shown in Table 1, 38.8% of the respondents in this study were in a supervisory position, and since there exists a presumption that supervisors will tend to give better scores, it is therefore important to examine the effect of position factor on the patient safety culture measurements. The average positive response rates for each of the 12 HSOPSC dimensions computed separately for the supervision group and the non-supervision group are summarized below:

(1) Supervisor/manager expectations and actions promoting safety: 84% for supervisors and 82% for non-supervisors,

(2) Organizational learning--continuous improvement: 84% for supervisors and 84% for non-supervisors,

(3) Teamwork within units: 93% for supervisors and 94% for non-supervisors,

(4) Communication openness: 59% for supervisors and 57% for non-supervisors,

(5) Feedback and communication about error: 61% for supervisors and 58% for non-supervisors,

(6) Nonpunitive response to error: 52% for supervisors and 41% for non-supervisors,

(7) Staffing: 42% for supervisors and 37% for non-supervisors,

(8) Hospital management support for patient safety: 60% for supervisors and 64% for non-supervisors,

(9) Teamwork across hospital units: 76% for supervisors and 71% for non-supervisors,

(10) Hospital handoffs and transitions: 54% for supervisors and 45% for non-supervisors,

(11) Overall perceptions of safety: 67% for supervisors and 64% for non-supervisors,

(12) Frequency of event reporting: 60% for supervisors and 56% for non-supervisors.

As we can see the average positive response rates for the supervision group were generally slightly higher than the rates for the non-supervision group, expect for the "Teamwork within unit" and the "Hospital management support for patient safety" dimensions, which the non-supervision group actually gave higher positive response rates. For all 12 dimensions, however, none of the differences in average positive response rate between the supervision group and non-supervision group were significant based on *t*-test.

### HSOPSC application in Taiwan

To justify the validity of using the HSOPSC on assessing patient safety culture in Taiwan, we used confirmatory factor analysis (CFA) in a structural equation model (SEM) to explore the fitness of applying HSOPSC in Taiwan. Table 3 summarizes the CFA results of applying HSOPSC on Taiwan data. The *X*^2 ^value of the CFA analysis is 63.65 (*p*-value = 0.002), implying that there is a discrepancy between the data in Taiwan and the hypothetical model. However, in using *X*^2 ^to test model fitness we must also consider sample size and the number of factors; a large sample size and large number of factors tend to decrease the fitness of the model [12]. The Normalized chi-square (NC) provides an alternative index of model fitness. As suggested by Hair et al. [13], an NC value larger than 1 and smaller than 5 should indicate fitness between the hypothetical model and sample data. The NC value for the data in this research is 1.872, thus indicates goodness of fit for the HSOPSC model with the Taiwan data. Furthermore, as shown in Table 3, almost all of the CFA statistics meet their respective goodness of fit criteria. The CFA results indicate acceptable model fitness between the hypothetical model of patient safety culture and the data in this study.

**Table 3:** Confirmatory factor analysis of applying HSOPSC in Taiwan.

Statistics	Values
Likelihood ratio *X*^2^	63.65
Normalized chi-square	1.872***
Root mean square error of approximation (RMSEA)	0.033***
Goodness of fit index (GFI)	0.986***
Adjusted goodness of fit index (AGFI)	0.960***
Expected cross-validity index (ECVI)	0.193***
Normalized fit index (NFI)	0.977***
Relative fit index (RFI)	0.956***
Incremental fit index (IFI)	0.989***
Tucker-Lewis index (TLI)	0.979***
Comparative fit index (CFI)	0.989***
Parsimony adjusted NFI (PNFI)	0.503***
Parsimonious goodness of fit index (PGFI)	0.430
Akaike information criterion (AIC)	151.6***
Critical number (CN)	601.0***

***Meet the goodness of fit criteria.

Table 4 shows the regression estimations for each dimension of the HSOPSC model. All 12 estimates are significant (*p*-value < 0.001) and are positive values, which means each dimension has its influence on patient safety culture. Of the 12 dimensions, nine have factor weights larger than 0.5, indicating that the nine factors have a direct effect on patient safety culture. The "Overall perception of patient safety" dimension has the highest factor weight, followed by the "Teamwork across hospital unit" dimension. The three dimensions that have factor weights less than 0.5 are "Feedback and communication about error", "Communication openness", and "Frequency of event reporting" (items 6, 7, and 8 in Table 4), which suggests that these factors have less influence on patient safety culture for the data in this study.

**Table 4:** Regression estimations for HSOPSC dimensions.

Dimension	Estimate	**S.E**.	Factor weights	*p*-value
1. Teamwork within units	1.000	----	0.505	----
2. Supervisor/manager expectations & actions promoting safety	2.153	0.211	0.667	< 0.001
3. Hospital management support for patient safety	1.538	0.163	0.683	< 0.001
4. Organizational learning -- continuous improvement	1.107	0.111	0.551	< 0.001
5. Overall perceptions of safety	1.895	0.190	0.704	< 0.001
6. Feedback & communication about error	0.486	0.139	0.142	< 0.001
7. Communication openness	0.529	0.096	0.239	< 0.001
8. Frequency of event reporting	0.673	0.184	0.150	< 0.001
9. Teamwork across hospital units	2.155	0.240	0.691	< 0.001
10. Staffing	1.700	0.182	0.619	< 0.001
11. Hospital Handoffs & transitions	2.014	0.230	0.630	< 0.001
12. Nonpunitive response to error	1.459	0.175	0.608	< 0.001

Table 5 shows the construct reliability (CR) values which are often used in conjunction with SEM. According to [13], CR values larger than 0.7 suggest good reliability, and values between 0.6 and 0.7 may be acceptable provided that other indicators of a model's construct validity are good. High construct reliability indicates that internal consistency exists. As shown in Table 5, many of the factors show good internal consistency. However, there are five factors that have CR values lower than 0.6, including "Staffing", "Overall perception of safety", "Communication openness", "Feedback and communication about error", and "Frequency of event reporting". There are several possible reasons for low internal consistency. One reason is that the factor structure of the HSOPSC model for these items might not fit the data well; another possible reason is that the sample size of the data might not be large enough to achieve consistency [13]. Also in Table 5, for discriminant validity, none of the 12 factors show high internal correlation, which provides evidence that the construct of patient safety culture is unique and captures some phenomena which is actually different in different dimensions.

**Table 5:** Construct reliability and internal correlation of HSOPSC.

factor	1	2	3	4	5	6	7	8	9	10	11	12
1	1.00											
2	0.282	1.00										
3	0.305	0.475	1.00									
4	0.233	0.363	0.392	1.00								
5	0.297	0.463	0.500	0.382	1.00							
6	0.068	0.106	0.115	0.087	0.112	1.00						
7	0.107	0.167	0.180	0.138	0.176	0.040	1.00					
8	0.065	0.101	0.109	0.083	0.106	0.024	0.038	1.00				
9	0.301	0.468	0.560	0.387	0.493	0.113	0.178	0.463	1.00			
10	0.258	0.402	0.435	0.332	0.424	0.097	0.153	0.093	0.429	1.00		
11	0.279	0.435	0.470	0.359	0.458	0.105	0.165	0.100	0.108	0.398	1.00	
12	0.242	0.377	0.409	0.312	0.397	0.091	0.143	0.087	0.402	0.346	0.373	1.00

CR	0.782	0.734	0.704	0.681	0.516	0.357	0.506	0.527	0.691	0.511	0.761	0.702

Note: 1 Teamwork within units; 2 Supervisor/manager expectation and actions promoting safety; 3 Hospital management support for patient safety; 4 Organizational learning--continuous improvement; 5 Overall perception of safety; 6 Feedback and communication abort error; 7 Communication openness; 8 Frequency of event reporting; 9 Teamwork across hospital units; 10 Staffing; 11 Hospital handoffs and transitions; and 12 Nonpunitive response to error.

## Discussion

The HSOPSC survey by AHRQ has been used to meet the increasing demand for patient safety culture assessment in the Western countries, especially in the US. In this study, we used HSOPSC to measure patient safety culture in Taiwan. Samples of 788 respondents from 42 hospitals across Taiwan were evaluated. Overall, the mean positive response rate for the 12 patient safety culture dimensions of the HSOPSC survey was 64%, slightly higher than the AHRQ data (61%). The results show that hospital staffs in Taiwan feel positively toward patient safety culture in their organization. The dimension that received the highest positive response rate was "Teamwork within units", which is similar to the results reported in US [6], Belgium [10], and Dutch [14]. On the other hand, the dimension that had the lowest percentage of positive responses was "Staffing", meaning that most of the respondents feel that staff allocation is not adequate to handle patient safety related workload. A similar finding was reported by Hellings and associates [10].

The HSOPSC survey results in this study reveal that the hospitals and health care organizations in Taiwan should have imperatives to:

◆ allocate staffs and working hours more adequately,

◆ develop a nonpunitive culture,

◆ focus on patient transfer and transition through the different units in the hospital,

◆ create an open communication atmosphere for reporting adverse events,

◆ establish an environment which helps staff report mistakes and errors spontaneously.

A positive safety culture will improve patient safety performance [15], which could help organization enhancing safety outcomes such as micro accident, self-report accident, safety behavior, and safety audit scores [16-19].

Most of the confirmatory factor analysis (CFA) indices indicate goodness of fit for applying the HSOPSC model of patient safety culture in Taiwan. Nevertheless, there are still some indices which do not support such a claim. This is not unexpected because questionnaires developed in one cultural setting normally cannot be translated and used directly in another cultural setting [20]. Our results show that there exist some discrepancies between the HSOPSC model and the data in Taiwan, particularly for the dimensions of "Feedback and communication about error", "Communication openness", and "Frequency of event reporting". Such discrepancies may be partly explained by the differences in organizational behavior between cultural settings, including management values, organizational commitments, leadership, and relationships within organizations [21,22].

The authors discovered several observations when applying HSOPSC measurements in Taiwan that might help explain some of the differences in patient safety culture between US and Taiwan. First, most of the respondents in Taiwan are shy of speaking up or asking questions when something which does not seem right has happened. Festinger [23] mentioned that in Chinese society, people care about others' thinking about their attitude and behavior. Chinese people tend to have strong social conformity in opinion or behavior, and also pursue interpersonal harmony. Harmonious relationships can maintain a stable social order that becomes the collective emotion of the Chinese [24]. Many Chinese think that communication openness might break the interpersonal harmony [25]. Therefore, when assessing the communication openness dimension of the HSOPSC in Taiwan or in Chinese society, concerns related to the internal psychological process of communication need to be addressed. For example, proper wordings should be used in the communication process in healthcare organizations during patient safety culture survey.

Second, many Taiwanese try to avoid discussing adverse events and errors directly and choose to stay silent. Previous studies found that Chinese society tends to be more collective than the Western society [26,27]. Yang and Yeh [28] mentioned that this kind of phenomenon is called "familial collectivism", which is the association of similarity between family and organizations. Adverse event reporting is an interaction between authority (as a father) and staff (as a child), which is considered a complicated relationship [29]. Chinese would tend to use metaphor and indirect methods to express their opinions, thus inhibiting them from expressing opinions freely. As stated in [30], culture plays a significant role in the interaction between supervisors and subordinates, and consequently culture differences may influence the degree of subordinates' supervisory commitment. In Taiwan, most employees tend to commit to the leaders [31]. When a staff member reports an error, he or she will become a 'whistleblower' and is seen as acting against the supervisor. Accordingly, the dimensions of "Communication openness" and "Frequency of event reporting" of patient safety culture have different implications for Taiwan and for the US.

Overall, HSOPSC has many strengths, such as good psychometric properties and comprehensive coverage of safety culture. However, patient safety culture studies must consider the diversity of cultures. Despite the progress of patient safety assessment that has been made in recent years, there remains a significant patient safety issue that has yet to be formally recognized and systematically addressed, namely, the issue of culture and its possible links to patient safety [32].

## Conclusions

There is currently a major effort to improve patient safety in many countries, and health care providers have been encouraged to assess the current state of their safety culture. Patient safety culture assessment tools provide an avenue to understand what staffs think and how they act towards patient safety. This paper discussed the application of the HSOPSC survey to assess the patient safety culture in Taiwan. A total of 788 staff members from 42 hospitals across Taiwan completed the survey. In general, hospital staffs in Taiwan feel positively toward patient safety culture within their organization. However, several dimensions of patient safety culture had low positive response scores, including "Staffing", "Nonpunitive response to error", and "Hospital Handoffs & transitions". Hospitals and health care organizations in Taiwan should address those issues while examining their quality of care. This paper also explored the differences in patient safety culture between the US and Taiwan using HSOPSC and gave explanations for some of the discrepancies found in the results.

The internal consistency of the data in this study was lower than that of the AHRQ data. The original AHRQ database is a large heterogeneous samples made up of many different health care organizations. Therefore, expanding the scale of the survey in Taiwan to cover more health care providers and practitioners is necessary for the future research. Moreover, patient safety culture measurements should consider the interaction between organizational and individual factors, which provide better understanding of group dynamics and individual attitudes of patient safety culture.

## Competing interests

The authors declare that they have no competing interests.

## Authors' contributions

ICC and HHL contributed to the conception of this paper, participated in its design and drafted the manuscript; ICC conceived the statistical methodology, provided the acquisition of data and performed statistical analyses. All authors have seen and approved the final version. ICC and HHL had full access to all of the data in the study and take responsibility for the integrity of the data and the accuracy of the data analysis.

## Pre-publication history

The pre-publication history for this paper can be accessed here:

http://www.biomedcentral.com/1472-6963/10/152/prepub
